# Physiology of the Pancreatic Secretion

**Published:** 1871-01

**Authors:** 


### PHYSIOLOGY OF THE PANCREATIC SECRETION.

M. Bernstein has published in the *Arbeiten aus der Physiologischen Anstalt zu Leipzig* some conclusions derived from experiments made, under the superintendence of C. Ludwig, upon the pancreatic secretion (*London Lancet*, November 19, p. 722). His views do not accord with those of Claude Bernard. He thinks a perfectly natural and healthy secretion can be obtained from dogs in which a permanent fistula of one of the ducts has been established; for he found the fluid thus obtained capable of converting starch into sugar, of emulsifying fat, and of digesting albumen, or, in other words, of converting it into peptone. The gland was found to be active in direct relation to the ingestion of food, the secretion increasing in quantity immediately after food had been taken, and attaining its maximum about two or three hours after a full meal, and then diminishing to the fifth or seventh hour, then slightly augmenting, and falling to its minimum at the fifteenth hour. Some of these results are similar to those already advanced by other authorities. The most interesting experiments were those instituted by M. Bernstein to investigate the influence of the nervous system on the pancreatic secretion. He found it greatly diminished in its flow when nausea was produced, while actual vomiting almost arrested it, the effect lasting for a considerable period. He also discovered, by irritating the pneumo-gastric, mechanically or otherwise, that any centripetal irritation of that nerve arrests the secretion,—that is to say, exerts an inhibitory influence on the activity of the gland.

